# Developing an Internet-Based Support System for Adolescents with Depression

**DOI:** 10.2196/resprot.2263

**Published:** 2012-12-12

**Authors:** Maritta Välimäki, Marjo Kurki, Heli Hätönen, Marita Koivunen, Maarit Selander, Simo Saarijärvi, Minna Anttila

**Affiliations:** 1 University of TurkuDepartment of Nursing ScienceTurkuFinland; 2 Southwest Hospital DistrictTurku University HospitalTurkuFinland; 3 Satakunta Hospital DistrictPoriFinland; 4 Pirkanmaa Hospital DistrictTampereFinland

**Keywords:** adolescent, Internet, depression, development, intervention, support

## Abstract

**Background:**

Depression is the most common mental health problem among adolescents. Despite policy guidance and governmental support to develop usable mental health services, there is still a lack of easily accessible and modern interventions available for adolescents in Finland’s majority official language.

**Objective:**

Our objective was to develop a user-friendly and feasible Internet-based support system for adolescents with depression.

**Methods:**

The Internet-based support system for adolescents with depression was developed. To create this new intervention, some examples of existing interventions were studied, the theoretical basis for the intervention was described, and the health needs of adolescents identified. As an outcome of the process, the results were combined and the content and delivery of a new intervention will be described here.

**Results:**

Six individual weekly Internet-based support sessions were delivered by a tutor over a 6-week period of time and developed to form an intervention called Depis.Net. This was an Internet-based support system for adolescents with depression tailored to improve self-management skills and increase awareness of their own well-being and mental health. The intervention was accessible via an electronic platform, which was secured and password protected for users. The intervention on the Depis.Net website consisted of elements identifying adolescents’ needs, and offering self-monitoring, access to health information and self-reflective written exercises. An educated nurse tutor gave written feedback to each adolescent via the electronic platform.

**Conclusions:**

An Internet-based support system for adolescents with depression was developed using a systematic approach with four steps. This was done to ensure that the intervention had a sound theoretical background and at the same time caters flexibly for the problems that adolescents commonly face in their daily lives. Its potential for adolescents visiting outpatient clinics will be evaluated in the next phase by means of a randomized controlled trial.

## Introduction

Mental health issues associated with adolescents have become a global public health problem [1,2]. It has been estimated that at least 20% of adolescents have some kind of mental health problem [3]. Depression is the most common mental health problem among adolescents with an estimated point prevalence of 4-6% [4]. In 2008, 8% of America’s adolescents had at least one major depressive episode during the past year [5]. Depression is associated with suicidal ideations and attempts [6] causing pervasive and prolonged functional impairment, morbidity and predicts psychopathology in adulthood [2,7]. Although most adolescents recover from their first depressive episode, the probability of recurrence is from 20% to 60% in 1 or 2 years after remission and 70% after 5 years [8].

A Cochrane review by Merry et al (2011) found some evidence that tailored and universal depression prevention interventions may prevent the onset of depressive disorders compared with no intervention [9]. In recent years, such interventions have been developed more systematically using information technology (IT). The development has made the Internet a source of mental health information [10,11]. Internet-based health interventions have also been used with adolescents for depression prevention, anxiety prevention [12], and depression disorders [13]. However, according to systematic reviews, among adolescents they are still much more rare than with adults [14,15]. A national survey of young Australians found that 71% of respondents rated websites and books for mental health information to be helpful, which was less than for counseling, which generally involves face-to-face meetings in mental health services [16].

Internet-based interventions are nowadays a way to offer behavioral intervention in an environment in which IT is available and the population is adapt at using it [17]. In most Western countries, including Finland, IT is an integral part of people’s lives and more than 80% of the people in this population are Internet users [18,19]. It is especially popular for adolescents’ daily communication [20] and computers are often placed in adolescents' bedrooms [21]. We therefore assumed that Internet-based interventions could be integrated into adolescents’ daily routines [22]. In Finland our new Health Care Act (2010/1326) highlights that health services should be near to users and also easily accessible for them [23]. In this paper we will describe a study in which we developed an Internet-based support system intervention for adolescents with depression who attend an adolescent outpatient clinic.

Previously, a variety of Internet-based devices have been developed and tested to support people with depression. These applications offer, for example, interventions treating depression online [24], increasing literacy among depression or reducing stigma and symptoms [25], Web-based cognitive behavioral therapy (CBT) [26], deducing symptoms using Internet support groups [27] or supporting people with depressive tendencies by a peer support social network service [28]. On the other hand, Internet-based methods [29] or other technical devices, such as mobile phones [30], have been less frequently used to help adolescents with depression. In 2012, Kauer et al [31] used mobile phones to facilitate self-monitoring for adolescents in their early stages of depression, while in 2009, Costin [32] used Health e-Cards to encourage help seeking. Further, van der Zanden and their group [13] developed an online group course for depression in adolescents and young adults in 2012.

However, devices to be used in depression have mostly been developed for speakers of English or for populous nations. Finnish is a relatively rare language compared to the fact that there are 5.4 million native speakers. Although Finnish adolescents generally nowadays have good English skills [33] helping them to integrate into the global world, services in Finnish, including mental health services has to be offered in Finnish language. Besides language, Finland is characterized by contrasting phenomena. For example, contradictions the education system is one of the best of the world and literacy in the skill of Finnish population is almost 100% [33]. The homogenous school system ensures that 97% of adolescents complete compulsory basic education [34]. At the same time, 5% of the age group between 15 and 29 years are excluded from the society, which means that they do not have a place to study, do not complete their education, or do not have a job [35]. Finnish adolescents also suffer from loneliness. It has been found that 9% of 8-9 graders had no close friends [36]. Other problems overshadowing Finnish adolescents include abuse of alcohol, which is related to low parental support and high parental alcohol abuse problems observed among depressed adolescent outpatients [37]. Other factors such as high divorce rates (2.5 divorces per 1,000 inhabitants) [38], high suicide rates for people ages 15-19 [39] also contribute to the high proportion of adolescents with depression compared to other countries. All these unique problems support a view that a culturally sensitive support system satisfying adolescents' needs in Finnish language should be developed.

## Methods

### Developing the Intervention

This intervention is an Internet-based support system designed for use in psychiatric adolescent outpatient clinics. In this intervention, we conducted measures in which the outcomes were synthesized and produced in the form of an Internet-based support system for adolescents with depression [40,41]. First, we reviewed the evidence for intervention development by searching the relevant literature on the topic. Second, we identified the most useful theories guiding intervention development. Third, we conducted interviews with professionals in social and health care services to gain a better perspective on adolescents’ world, problems, or needs and possible solutions to these problems. Finally, we co-operated with nursing staff to ensure that the intervention would be adopted as part of the existing treatment environment and procedures, and that these newly developed interventions would benefit the adolescents, professionals, and services involved.

### Reviewing the Evidence-Based Knowledge

Internet-based interventions are based on a variety of treatment ideologies and theoretical backgrounds, such as CBT [14], self-help [42], or problem-solving therapy [12]. The findings of a meta-analysis of the effectiveness of Internet-based psychotherapeutic interventions provide strong support for the adoption of online psychological interventions [43]. There are also indications that IT is superior to waitlist and control assignments and the effects of IT are equal to therapist-delivered treatment across anxiety disorders [44]. Moreover, adherence to and satisfaction with computerized CBT interventions is good among patients despite the significantly reduced amount of contact with the clinician [45]. However, dropout rates are considerable [45] even though comparable to those reported in other psychological therapies [46]. The most common reason for dropout is that the participants were too busy or have a change in circumstance, with only a few trials reporting that the treatment was not useful as the reason for drop outs [46]. For IT to be effective, it must be technically reliable and robust, well accepted by clients and health care professionals, capable of producing services equivalent in quality to face-to-face consultations without undue disruption to practice patterns [47].

A number of Internet-based interventions for adult anxiety and depression have been developed and implemented, but there are fewer interventions for adolescents [14,15,43,48]. In Australia a randomized controlled trial study conducted by Calear et al examined the effectiveness of the MoodGym in a large sample of adolescents (N = 1477) in a school environment with the aim of targeting both anxiety and depressive symptoms. However, the effects on depressive symptoms were effective only in male participants in the intervention group at post-intervention and at 6-month follow up [49].

In addition to the present study, we found 4 registered clinical trials focusing on the use of IT among adolescents with depression. These registered studies were searched from the Clinical Trials Search Portal (search terms were adolescent AND depression AND information technology OR computer). This portal provides access to a central database containing the trial registration data sets provided by the most common registries. All these 4 studies [50-53] focus on CBT using IT. These studies targeted 546 adolescents with mild to severe depressive disorder. Interventions under investigation include self-directed programs and programs where adolescents receive counseling (Table 1). Based on this information, the knowledge base regarding the use of CBT will be strengthened. However, the present study investigated the use of IT as a method to increase self-management among adolescents with depression. The method is intended to support and strengthen the delivery of the treatment, not to replace it.

**Table 1:** Description of the registered randomized controlled trials considering the use of information and communication technology among adolescents with depression.

Intervention	Target group	Estimated enrollment	Country	Title, register, identification, and status
Computer guided, CBT delivered by a clinician-administered telephone intervention	Adolescents with major depressive disorder	150	US	Information Technology Enabled Treatment of Adolescent Depression ClinicalTrials.go NCT01582581 Recruiting
Stressbusters, computerized CBT program	Adolescents with low mood or depression	96	UK	A feasibility study and pilot trial of computerized CBT for depression in adolescents ISRCTN ISRCTN31219579 Recruiting
The computer-administered self-directed program is based on CBT	Adolescents with mild to moderate depressive symptoms	200	New Zealand	Youth e-therapy - Evaluation of a computerized CBT self-help program for adolescents with mild to moderate depression ANZCTR ACTRN12609000249257 Closed
A computer resource with a CBT-based guided self-help intervention with minimal supervision from a school guidance counselor or clinician	Adolescents with mild to moderate depression	100	New Zealand	Computerized delivery of a CBT-based self-help intervention for the treatment of depression in adolescents: development and pilot study ANZCTR ACTRN12606000142538 Recruiting

### Theoretical Framework of the Intervention

As a theoretical framework we chose the self-determination theory (SDT) [54,55], which gave us guidance on how to support adolescents’ natural or intrinsic tendencies to behave in an effective and healthy way. SDT focuses on the degree to which an individual’s behavior is self-motivated and self-determined. In the context of an adolescent self-management intervention, the theory would suggest that adolescents’ motivation to manage their own situations and well-being depends on their intrinsic motivation, which can be supported by increasing their positive feeling of autonomy, competence, and being connected [56,57]. In addition, a sense of connectedness should represent interpersonal acceptance and closeness. Thus, guided by the SDT we decided to plan our intervention so that at the beginning the adolescents should have a chance to identify and describe their own perceptions of their situations and possible concerns to ensure that adolescent’ inner motivation and needs are identified from the start.

By so doing we were also more aware of what kind of information and support adolescents need to resolve in their individual situations. To support adolescents’ competence and sense of autonomy, we focused on several areas, including knowledge-based awareness, self-monitoring, self-awareness (moods, feelings), social relationships (networking, friends, family relations), and life style (habits, circadian rhythm). In addition, a need for relatedness was supported by a person offering interpersonal acceptance, who is not too far away and close enough to be present and connected to the adolescent whenever needed, and who imparts a caring atmosphere without being too officious. The intervention then consisted of a variety of elements: identification of adolescent needs, self-monitoring, access to health information, and self-reflective written exercises. We also offered weekly positive feedback to promote feelings of competence. Positive feedback was previously shown to be effective in enhancing intrinsic motivations while negative feedback impairs them [58].

### Interviews with Social and Health Care Professionals

To ensure that the support system intervention to be developed would satisfy the needs of suffering adolescents in their daily work (adolescent workers, police officers, staff from the emergency services and the family center, special teachers at school, and from the adolescent work of the Church), semi-structured interviews with only one theme were conducted in 2006. During the interviews, these 2 questions were posed to 17 participants: (1) What are the main problems that adolescents aged 15-17 years encounter? (2) What support do these adolescents need from a professional standpoint? The interview responses were analyzed using inductive content analysis [59].

The analysis revealed 5 key problems related to adolescent care. First, there was a lack of structure in adolescents’ lives; they have no daily routines and rules and they have problems to cope with. They have irregular daily routines; they wake up late, have irregular meal times or do not have meals at all. Second, adolescents are obsessed with a single interest and activity. Third, many adolescents have issues with drug and alcohol abuse. Fourth, adolescents make no effort to excel in school. Finally, professionals are concerned about the interference and harassment associated with text messaging, email and Internet use. In particular, adolescents may not be aware that insults and threats communicated in text-messaging and emails are as abusive and threatening as other forms of such insults.

In light of the problems identified by the social and health care professionals, adolescents must be targeted with information about the importance of daily routines and rules, for example, how important they are to their physical and mental health. Adolescents need information about healthy sleep patterns and regular and healthy food for their health and well-being. Adolescents need information about the choices after comprehensive schooling, how these affect the rest of their lives, and the way they will be placed in society. Further, adolescents need information on good manners, how to interact well with other people. Additionally, they need information on legal aspects and rules on the use of modern technology and the Internet. Besides professionals, adolescents were also interviewed about their main informational needs and the topics in the support system were based on these themes.

### Adaptation to the Intervention in a Specific Treatment Context

The intervention was to be adapted into the practice of 2 psychiatric outpatient clinics in southern Finland. The clinics serve over 2 million people in their catchment areas and offer specialized care for adolescents referred for psychiatric outpatient care. The clinics are specialized in the examination and treatment of adolescents less than 20 years old who are likely to be suffering from depression. There were in total 14 nurses, 8 psychologists, 7 social workers and 8 physicians working in these clinics.

To ensure that the intervention would be well integrated in the outpatient clinic an adaptation process was planned. It was based on the Technology Acceptance Model (TAM) [60], which posits that perceived usefulness of an intervention influences the perceived ease of use. These 2 components then determine an individual's intention to use and actual usage of a system. In practice, however, constraints in ability, time, environment, organization, and unconscious habits will limit intentions to act [61]. Therefore, we first arranged a series of educational sessions introducing background knowledge of Internet-based intervention use as a part of clinical care. Second, we conducted interviews to ascertain how useful nurses thought the intervention could be. Third, to ensure ease of use, the nurses were involved in different parts of the development process. They produced information on practical constraints and how these could be resolved. The aim of these actions was to support actual system use. Moreover, to promote nurses’ intervention use, a manual for the intervention was developed including detailed descriptions of the background and principles of the intervention, as well the practical guidance on how to use the Depis.Net program.

### The Intervention

**Figure 1:**
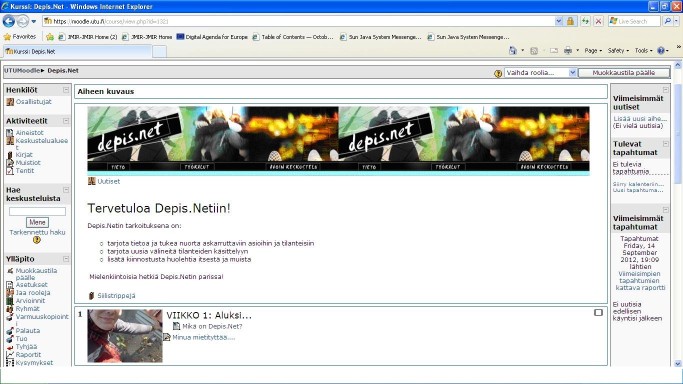
Depis.Net website.

**Figure 2:**
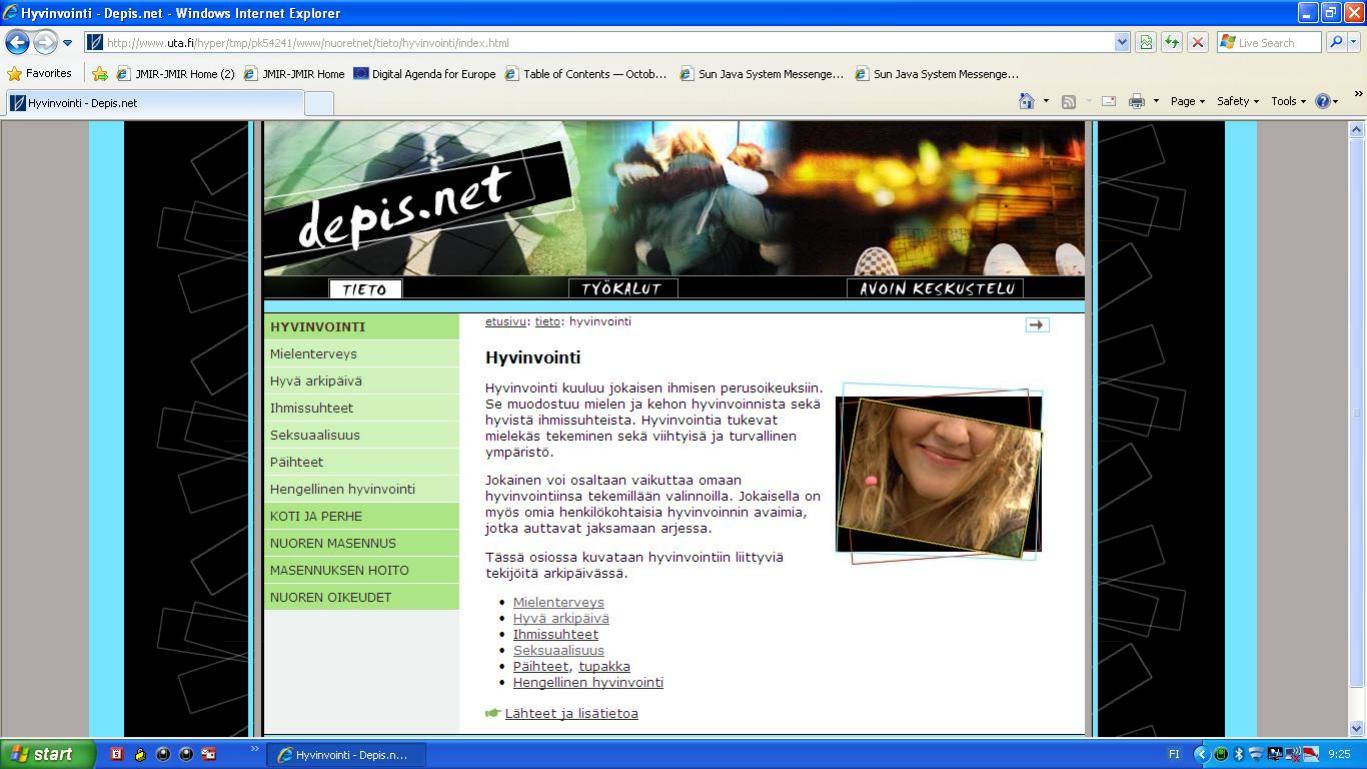
Different themes of the intervention on the Depis.Net website.

As an outcome of the systematic development process an intervention called ‘Depis.Net’ was developed (Figure 1). At this stage, it was developed for adolescents who had received a referral to an outpatient clinic. In a wider perspective, this can be used for adolescents with suspected depression or anxiety disorder, for example at school. This Internet-based support system intervention was developed to support adolescents’ self-management, and to increase their awareness of issues associated with well-being and mental health. It was accessible via an electronic platform, which was secured and password protected for users. The intervention consisted of the following basic elements: identification of adolescent’s concerns, self-monitoring, access to health information (Depis.Net website) and self-reflective written exercises . The intervention included 6 individual sessions to be delivered over a 6-week period. There was an introductory session, followed by sessions that focused on a different topic per week: (1) well-being, (2) home and family, (3) adolescent’s rights and responsibilities, (4) adolescent depression, and (5) treatment of adolescents’ depression (Figure 2). Adolescents had one week to process each topic. To ensure that the intervention was targeted at adolescents and user-friendly, volunteer adolescents were encouraged to take the photos included in the program, to design its external appearance, write the poems included in the program, and give feedback on the informational content of the support system. The content, exercises, and self-monitoring methods of each session are described in Table 2.

**Table 2:** Themes, exercises, and self-monitoring activities in the Internet-based support system.

Week	Theme	Exercises and self-monitoring
Introductory session	Introduction for independent working	Describe the focused problem
Week 1	Well-being	Working with focused problem
		Working with well-being theme
		Mood diary
		Question corner
Week 2	Home and family	Working with focused problem
		Working with home and family theme
		Network map
		Depression Scale (BDI-21)
		Mood diary
		Question corner
Week 3	Adolescent’s rights and responsibilities	Working with focused problem
		Working with adolescent's rights and responsibilities theme
		Mood diary
		Question corner
Week 4	Adolescent depression	Working with focused problem
		Working with adolescent depression theme
		Rating sleeping diary
		Mood diary
		Question corner
Week 5	Treatment of adolescent’s depression	Working with focused problem
		Working with treatment of adolescent’s depression theme
		Assessment of sleeping diary
		Depression scale (BDI-21)
		Mood diary
		Question corner

The intervention was implemented in psychiatric outpatient clinics by trained tutors. Before the 6-week intervention there was a face-to-face discussion between each adolescent and a tutor. The aim was to provide basic information on the Depis.Net intervention. During the introductory session, the tutor supported the adolescent to write down his/her focused concern or problem so that he/she could work with it throughout the 6-week period from different angles. An intervention was followed by weekly accessible health information, self-monitoring exercises, self-reflective diaries and other exercises via a website in which the adolescent reflects his/her own life situation. Adolescents returned their diaries each week and the tutor offered support and feedback via the website. Supportive text messages were sent to the adolescents once a week before the next topic started if they had not already visited it. Moreover, in case of serious concern based on adolescents’ written texts and exercises (eg, suicidal indication), the outpatient clinic was contacted immediately. The regular and need-based support provided is described in Table 3 and Table 4.

**Table 3:** Regular support provided in the Internet-based support system.

Regular weekly support	Aim
Text message to continue to the next theme	To encourage use of the support system and communicate its progress
Individual feedback on Moodle	To encourage working on the support system
Reflecting on the participant’s work in the support system when meeting the therapist face to face in the outpatient clinic	To integrate the theme and adolescent’s work into the care process

**Table 4:** Need-based support provided in the Internet-based support system.

Needs-based support	Aim
Supportive text message if an adolescent had not done any weekly exercises	To motivate and avoid drop-out
Contact to the outpatient clinic in case of concern about adolescent's severe symptoms such as suicidal ideation	To ensure adolescent’s safety and maintain quality support system

## Discussion

This paper describes the process of developing an Internet-based support system intervention for adolescents. We included 4 steps to develop a need-based intervention for adolescents with depression treated in outpatient psychiatric clinics in Finland.

The theoretical framework chosen for the intervention, SDT [54,55], supported our intention to use such a method for several reasons. In SDT Ryan and Deci [56,57] posit 3 innate basic needs motivating human behavior: autonomy, competence and relatedness. First, autonomy refers to the feeling of being responsible for one's own actions. This supported our intention to use such a method, because it allows adolescents to work independently in the system and to discuss their achievements later on with their support person. Second, competence refers to being effective in dealing with the environment in which a person finds him/herself. In many cases, adolescents have poorer self-esteem and their sense of self-competence is weak [62]. Therefore it was important that through intervention activities, they should feel that they could cope with the assignments without stress. Third, relatedness represents interpersonal acceptance, closeness, and the universal desire to interact, be connected to, and caring for others. However, there are individual differences in the extent to which basic needs are satisfied or thwarted [58,59]. This convinced us that instead of a stand-alone online system, adolescents need a human interaction component in the system to feel support. [62].

TAM model was selected to structure the implementation process, understand acceptance, and use of IT among adolescents [60]. Moreover, by integrating the development process according to the TAM model, we were able to make the support system as usable and as acceptable as possible.

It is a limitation of the development process that no systematic literature review was conducted. The process of the systematic review is not appropriate in all literature reviews and therefore we considered using recently published reviews and study reports [40,41] for this. Moreover, even if adolescents were not actively involved throughout the whole development process, their opinions on content and layout were expressed in expert groups and considered in all phases of the Depis.Net intervention. The aim was to ensure that the intervention responded to the adolescent’s needs and was perceived to be attractive.

The strength of the intervention is that it was developed on the basis of an established theoretical framework. The development process was conducted through several steps even though it was challenging to integrate different forms of information. The aim was to ensure that the intervention had a sound theoretical background yet flexible in the considerations for the problems that adolescents currently face in their daily lives. Although a number of Internet-based interventions have been developed and implemented for adult anxiety and depression, such interventions are scarce among adolescents with depression [14,15,43,48]. Therefore the recent literature led us to believe that an intervention of this sort would have great potential [22,46,50]. Moreover, integrating professionals’ viewpoints into the existing knowledge base ensured that the intervention addressed topical concerns.

The potential of the intervention developed needs to be investigated, especially because there is still diversity in what is known about the effectiveness of these interventions [22] and a lack of consensus as to the effective components of these interventions [63]. Thus the potential of the Depis.Net intervention for adolescents attending outpatient clinics will be evaluated in the next phase by means of a randomized controlled trial (NCT00054925). As a practical consideration, in order for an intervention for adolescent depression to be truly effective it must be accessible and desirable in a natural setting. It is important that adolescents make the decision to participate in it and make a commitment to follow an intervention on their own. Moreover, Internet-based interventions must be continuously maintained. The intervention will be further developed after evaluation of the results and feedback obtained in future studies.

## Abbreviations

CBT: cognitive behavioral therapy
IT: information technology
SDT: self-determination theory
TAM: technology acceptance model
